# Endoscopic coblation-assisted and partial arytenoidectomy for infants with idiopathic bilateral vocal cord paralysis

**DOI:** 10.1097/MD.0000000000028593

**Published:** 2022-01-28

**Authors:** Letian Tan, Chao Chen, Qi Li

**Affiliations:** Department of Otolaryngology-Head and Neck Surgery, Children's Hospital of Fudan University, Shanghai, China.

**Keywords:** arytenoidectomy, coblator, endoscopic, idiopathic bilateral vocal cord paralysis, infants

## Abstract

To review our experience with endoscopic coblation-assisted and partial arytenoidectomy (ECPA) in treating idiopathic bilateral vocal cord paralysis (BVCP).

A retrospective analysis of thirty-three infants (19 boys and 14 girls, aged 1–10 months) with idiopathic BVCP undergoing ECPA was performed. The therapeutic process and outcomes (surgical success, swallowing function, and voice) were reviewed. The follow-up period was >33 months.

Among the thirty-three infants with idiopathic BVCP, surgery was successful in twenty-nine cases but failed in four cases. Twenty-one, nine, and three patients underwent right, left, and bilateral ECPA, with surgical success rates of 90.5%, 100.0%, and 33.3%, respectively. In addition, four and six cases were combined with subglottic stenosis (SGS) and laryngomalacia, respectively. The surgical success rates of BVCP alone and BVCP+ other airway abnormalities were 95.6% and 70.0%, respectively. During the follow-up, five infants had slight difficulty swallowing, 12 infants had partial or complete recovery movement of at least one vocal cord with satisfactory voice outcome, and five infants had early granuloma formation, which disappeared spontaneously.

ECPA appears to be a promising alternative to tracheostomy and initial management in infants with idiopathic BVCP who are free of other airway abnormalities.

## 1 Introduction

Bilateral vocal cord paralysis (BVCP) is a rare condition, with an estimated incidence of 0.75 cases per 1 million births per year,^[1]^ which may be traumatic (delivery, surgery, etc.), neurological, and idiopathic. When no specific cause was found, the condition was considered idiopathic.^[2]^ BVCP is life-threatening, resulting in airway obstruction, aspiration, swallowing disturbances, and voice changes.^[3]^ The primary goal of treatment is to provide an adequate airway for ventilation while minimizing adverse effects such as aspiration and dysphonia. Therefore, tracheostomy is the gold standard to achieve this goal for some time, preserving an acceptable respiratory function and laryngeal architecture but has significant morbidity and even mortality in pediatric patients.^[4]^ More than 50% of pediatric patients present with spontaneous recovery of vocal cord paralysis in the first 12 months of life^[5]^; hence, the management of this condition has evolved to a more conservative approach, developing different management strategies and surgical alternatives to avoid tracheostomy. Endoscopic total arytenoidectomy is a traditional procedure for BVCP, defined in the middle of the 20th century.^[6]^ Nowadays, endoscopic partial arytenoidectomy is preferred as it attains a good airway with better voice preservation and less aspiration compared with total ablation.^[3]^ Lasers can be an effective tool, but intraoperative bleeding can sometimes be encountered, and postoperative granulation tissue and collateral heat injury are possible.^[7]^ Since it is safe and efficient, a coblator has been employed to perform partial arytenoidectomy in some studies.^[8,9]^

In this study, we describe the utility and outcomes of endoscopic coblation-assisted and partial arytenoidectomy (ECPA) in infants with idiopathic BVCP and discuss the feasibility and efficacy of this procedure.

## 2 Materials and methods

### 2.1 Patients

Infants with idiopathic BVCP who underwent ECPA treatment at the Infants’ Hospital of Fudan University between January 2016 and December 2018 were included in the retrospective analysis. They were diagnosed with BVCP using a flexible laryngoscope while awake, and underwent neuropediatric clinical examination, cardiac ultrasonography, and cerebral magnetic resonance (MR) to determine the type of paralysis. Cases of iatrogenic, traumatic, neurological, and tumor-induced were excluded, and only idiopathic BVCP was included. To confirm a combined airway abnormality, infants underwent microlaryngobronchoscopy (MLB) detection under general anesthesia. The following data were reviewed from the medical records: age, sex, disease status, surgical interventions, outcomes, and adverse events. All infants were intubated preoperatively so that the modified barium swallow (MBS) test could not be performed; instead, the MBS test was performed for each child postoperatively. This study was approved by the Ethics Committee of the Infants’ Hospital of Fudan University.

### 2.2 Surgical intervention and postoperative management

The ECPA was performed under general anesthesia with the patient spontaneously ventilating, and microlaryngobronchoscopy detection was performed to expose the whole glottis. The tip of the coblator was placed on the surface of the arytenoid to ablate the overlying mucosa, but not the arytenoid itself. When the membrane of the arytenoid was exposed, the procedure was completed. All infants were nasotracheal intubated and sedated in the intensive care unit for 3–5 days. The endotracheal tube used was one size smaller than the age-appropriate tube. During this period, all infants received anti-reflux medication with proton inhibitors, analgesics, intravenous antibiotics, and methylprednisolone (2 mg/kg/day). If extubation failed, ECPA was performed on the contralateral arytenoid, and if it failed again, tracheostomy was performed.

### 2.3 Postoperative evaluation and follow-up

According to the “Outcome Measures for Pediatric Laryngotracheal Reconstruction: International Consensus Statement” published in 2018,^[10]^ the functional outcomes of the surgery in terms of breathing, swallowing, and voice were evaluated and recorded. Surgical success was defined as the ability to avoid tracheostomy, even with limited exercise tolerance. Surgical failure was defined as the inability to avoid tracheostomy. Respiratory outcomes included extubation or tracheostomy, and exercise tolerance. All patients underwent the MBS test to identify swallowing function, and voice evaluation was based on clinical observations. Anatomic follow-up was based on awake flexible laryngoscopy (1st postoperative month, 3rd month, 6th month, and later twice per year), and the grade of vocal cord movement recovery was recorded. Voice evaluation was based on clinical observation^[11]^:

1. normal voice;
2. mild dysphonia: hoarse voice with some difficulties being heard or understood in a loud environment;
3. moderate dysphonia: weak voice or ventricular band phonation with easy fatigability;
4. severe dysphonia: breathy voice with difficulty in communicating;
5. Aphonia.

## 3 Results

### 3.1 Demographic features

Thirty-three infants with idiopathic BVCP were included in this study: 19 cases were boys and 14 cases were girls, aged from 1 to 10 months (median age, 4 months). All infants had severe dyspnea and underwent tracheal intubation. Four and six infants were combined with grade I subglottic stenosis (SGS) with Cotton–Myer and laryngomalacia, respectively, and the remaining 23 infants had idiopathic BVCP alone.

### 3.2 Outcome of surgical treatment

Twenty-one infants underwent right ECPA, nine patients underwent left ECPA, and three patients underwent bilateral ECPA. No major perioperative or postoperative complications were observed. As shown in Table 1, the surgical success rates of right, left, and bilateral ECPA were 90.5%, 100.0%, and 33.3%, respectively. In total, 29 (87.9%) cases were surgically successful, and four (12.1%) were surgical failures. Among the failed cases, two underwent bilateral ECPA and two underwent right ECPA. The mean duration of intubation was 3.6 days.

**Table 1:** ECPA outcome of infants with idiopathic BVCP.

	Surgical success		
	Full exercise tolerance	Limited exercise tolerance	Surgical fail
Right ECPA (n = 21)	14 (66.7%)	5 (23.8%)	2 (9.5%)
Left ECPA (n = 9)	8 (88.8%)	1 (11.1%)	0 (0.0%)
Bilateral ECPA (n = 3)	0 (0%)	1 (33.3%)	2 (66.7%)
Total (n = 33)	29 (87.9%)		4 (12.1%)

BVCP = bilateral vocal cord paralysis, ECPA = endoscopic coblation-assisted and partial arytenoidectomy.

We further explored whether surgical success was associated with other airway abnormalities. The surgical success rates of BVCP alone, BVCP + laryngomalacia, and BVCP + SGS were 96%, 83%, and 50%, respectively (Table 2). The surgical success rate of BVCP + laryngomalacia and BVCP + SGS (7/10, 70.0%) was lower than that of BVCP alone (22/23, 95.6%), suggesting that the effect of ECPA on idiopathic BVCP infants is poorer when combined with other airway abnormalities.

**Table 2:** ECPA outcome of infants with idiopathic BVCP combined with/without SGS or laryngomalacia.

	Surgical success		
	Full exercise tolerance	Limited exercise tolerance	Surgical fail
BVCP + SGS (n = 4)	0 (0.0%)	2 (50.0%)	2 (50.0%)
BVCP + LM (n = 6)	5 (83.3%)	0 (0.0%)	1 (16.7%)
BVCP alone (n = 23)	17 (73.9%)	5 (21.7%)	1 (4.3%)

BVCP = bilateral vocal cord paralysis, ECPA = endoscopic coblation-assisted and partial arytenoidectomy, LM = laryngomalacia, SGS = subglottic stenosis.

All infants were fed orally after the ECPA intervention. However, based on the postoperative MBS test, five infants were aspirated on thin liquid and were clinically symptom-free in oral feeding by feeding therapy and thickening of the diet. The follow-up time ranged from 33 to 48 months. During the follow-up, 12 patients (36.0%) had partial or complete recovery of at least one vocal cord with a satisfactory voice outcome, with an average duration of 18 months. Furthermore, there were five cases of early granuloma formation during follow-up, which disappeared spontaneously, and no excessive granuloma formation was observed in any patient.

## 4 Discussion

Traditionally, tracheostomy was an appropriate choice, but advances in endoscopic laryngeal surgery have enabled otolaryngologists to avoid tracheostomy and its associated morbidity. Endoscopic techniques often require a higher degree of expertise and specialized equipment, whereas tracheostomy is the standard operation for most otolaryngologists. However, when long-term costs, most notably tracheostomy care, are factored in, endoscopic management is not only more effective, but also less expensive.^[4]^ Additionally, quality of life issues pertaining to voice, appearance, and daily management of a tracheostomy tube; therefore, alternatives to tracheostomy are valuable.

Pediatric cases, especially infants and neonates, make the management of BVCP more demanding and impending. Many endoscopic procedures have been debated in pediatric patients, such as posterior cordotomy,^[12]^ arytenoidopexy,^[13]^ vocal cord lateralization,^[14]^ and anterior–posterior cricoid split,^[15,16]^ but there is still no ideal solution for all BVCP problems. In the past 10 years, a few studies have focused on the combination of coblator and arytenoidectomy in the management of BVCP in adults and older infants.^[8,17]^ To our knowledge, this is the first report to discuss this procedure with regard to infants. Arytenoidectomy is generally performed by removing the arytenoid cartilage together with the mucosa, which carries the risk of granuloma and scar formation.^[3]^ However, by only removing the overlying mucosa and keeping the membrane of the arytenoid, there is minimal granuloma and scar formation. In our cases, there were five cases of early granuloma formation during follow-up, which disappeared spontaneously, and no excessive granuloma formation was observed in any patient. Coblator, which can rapidly remove tissue at low temperatures without significant bleeding, has dramatically facilitated this ablative procedure and reduces collateral tissue damage. In addition, based on our experience, the coblator equipment is more accessible than the aforementioned surgical procedures, and the technique is less fiddly, which may help to popularize this procedure in infants, even in neonates.

In our series, bilateral ECPA (33.3%) had a lower success rate than the right (90.5%) and left (100.0%) ECPA. However, we did not consider bilateral ECPA as less effective than unilateral ECPA as a more morbid patient has a higher risk of failure in unilateral ECPA; thus, it is more likely to undergo bilateral ECPA. For example, two of the three patients in this study who failed in bilateral ECPA had additional airway anomalies. Since this study is a small sample, more cases and evidence are required to support our opinion.

In this study, ten infants had additional airway abnormalities, with laryngomalacia and SGS being the most common. Our results demonstrated that patients with additional airway anomalies had a lower success rate (70.0%) than those with BVCP alone (95.6%). Although its impact could not be quantified, we consider the association with additional airway anomalies as a risk factor for ECPA, emphasizing that otolaryngologists should be cautious and vigilant in dealing with these patients. In addition, alternative procedures that could solve both BVCP and additional anomalies simultaneously should be considered; for instance, an anterior–posterior cricoid split may be more appropriate for patients with both BVCP and SGS. Thus, further studies are ongoing to improve patient selection and patient outcomes.

The success rate of diverse endoscopic procedures varies in the literature. Yilmaz reported that 56 out of 64 patients (88%) did not report dyspnea in their daily life after endoscopic partial arytenoidectomy using a CO_2_ laser.^[3]^ In Rutter's study, surgery was considered successful in 14 of 19 patients (74%) regarding the anterior–posterior cricoid split.^[15]^ Basterra et al reported that 17 of 18 patients (94%) were asymptomatic with respect to breathing after posterior cordotomy using microelectrodes.^[18]^ In a recent study, Hu reported that 13 of 14 patients (93%) underwent removal of the tracheal tube safely within 1 month after coblation-assisted partial arytenoidectomy.^[8]^ In this study, with an overall success rate of 88%, we consider that ECPA is a reasonable and acceptable procedure. Our findings illustrated that ECPA had a promising outcome in swallowing function and voice, attributed to the minor excision range and preservation of the height of the arytenoid. Another advantage of ECPA is that it provides a chance for revision if the primary procedure fails, alternative procedures can be easily performed, either endoscopic or open, as a revision. Therefore, it may be a sound practice to perform ECPA initially in idiopathic BVCP cases.

## 5 Conclusion

Based on our experience with thirty-three cases, ECPA appears to be a promising alternative to tracheostomy and initial management in infants with idiopathic BVCP who are free of other airway abnormalities.

## Acknowledgments

None.

## Author contributions

**Conceptualization:** Chao Chen.

**Data curation:** Chao Chen.

**Investigation:** Qi Li.

**Methodology:** Chao Chen.

**Software:** Qi Li.

**Supervision:** Chao Chen.

**Validation:** Chao Chen.

**Writing – original draft:** Letian Tan.

**Writing – review & editing:** Letian Tan.
